# Epidemiology of acute respiratory infections in children - preliminary results of a cohort in a rural north Indian community

**DOI:** 10.1186/s12879-015-1188-1

**Published:** 2015-10-26

**Authors:** Anand Krishnan, Ritvik Amarchand, Vivek Gupta, Kathryn E. Lafond, Rizwan Abdulkader Suliankatchi, Siddhartha Saha, Sanjay Rai, Puneet Misra, Debjani Ram Purakayastha, Abhishek Wahi, Vishnubhatla Sreenivas, Arti Kapil, Fatimah Dawood, Chandrakant S. Pandav, Shobha Broor, Suresh K. Kapoor, Renu Lal, Marc-Alain Widdowson

**Affiliations:** Centre for Community Medicine, All India Institute of Medical Sciences, New Delhi, 110029 India; The INCLEN Trust International, New Delhi, 110020 India; Centers for Disease Control and Prevention, Atlanta, GA 30333 USA; Department of Community Medicine, Velammal Medical College Hospital and Research Institute, Madurai, 625009 India; Department of Biostatistics, All India Institute of Medical Sciences, New Delhi, 110029 India; Department of Microbiology, All India Institute of Medical Sciences, New Delhi, 110029 India

**Keywords:** Acute respiratory infections, Burden, Children, Cohort, Developing countries, Epidemiology, Pneumonia

## Abstract

**Background:**

Despite acute respiratory infections being a major cause of death among children in developing countries including India, there is a lack of community-based studies that document its burden and aetiology.

**Methods:**

A dynamic cohort of children aged 0–10 years was established in four villages in a north Indian state of Haryana from August 2012 onwards. Trained health workers conducted weekly home visits to screen children for acute respiratory infection (ARI) defined as one of the following: cough, sore throat, nasal congestion, earache/discharge, or breathing difficulty. Nurses clinically assessed these children to grade disease severity based on standard age-specific guidelines into acute upper or lower respiratory infection (AURI or ALRI) and collected nasal/throat swabs for pathogen testing.

**Results:**

Our first year results show that ARI incidence in 0–10 years of age was 5.9 (5.8–6.0) per child-year with minimal gender difference, the ALRI incidence in the under-five age group was higher among boys (0.43; 0.39–0.49) as compared to girls (0.31; 0.26–0.35) per child year. Boys had 2.4 times higher ARI-related hospitalization rate as compared to girls.

**Conclusion:**

ARI impose a significant burden on the children of this cohort. This study platform aims to provide better evidence for prevention and control of pneumonia in developing countries.

## Key messages

1. An AIIMS-CDC Surveillance platform at Ballabgarh for Acute Respiratory Tract Infections (SuBhARTI) has been established to study the epidemiology of Acute Respiratory Infections in a rural north Indian community.
2. The first year results of the open cohort of 2859 children aged 0–10 years with weekly house visits showed a high burden of acute respiratory infection in children.
3. There was a significant gender difference skewed towards males in the burden and health seeking.

## Background

Acute respiratory infections (ARI) are a major burden to child health in developing countries like India [1, 2]. ARI, mainly of lower respiratory tract, are the leading cause of death among children under five years of age in such countries [3-5], resulting in nearly 1.9 million childhood deaths per year, of which 20 % are estimated to occur in India [6, 7]. Worldwide, about 85–88 % of ARI episodes are Acute Upper Respiratory Infections (AURI) while the remaining are Acute Lower Respiratory Infections (ALRI) [8–10]. In the most recent estimate of ALRI associated mortality in India, pneumonia was held responsible for 369,000 deaths (28 % of all deaths) among those 1–59 months, making it the single most important killer in this age group [11]. ARIs also impose a significant economic burden on health systems and individual families in developing countries. We recently estimated that among children aged < 5 years, the median direct cost of ARI was US$135 in private and US$54 in public institutions [12].

Studies from Bangladesh and Pakistan, estimate the average cost of treatment for a single episode of pneumonia as US$ 13 for outpatient care and US$ 71 to US$235 for severe hospitalized pneumonia. It was also estimated that 75 % of the families spent more than half of their total monthly expenditure on hospitalization [13, 14].

This burden of respiratory infections, both globally and in India, can be reduced by many proven effective strategies. The Global Action Plan for Prevention and Control of Pneumonia (GAPP) prioritizes them as case management, vaccination, prevention and management of HIV infection, improvement of nutrition and breastfeeding, reduction of low birth weight, and control of indoor air pollution [15]. Identification of risk factors and etiological agents of pneumonia were among the top ten research priorities by an Expert Group on Childhood Pneumonia in 2011 [16].

A recent systematic review for advocacy and action on pneumonia in India identified the lack of evidence on epidemiology and etiology of pneumonia as important barriers to effective planning and implementation of preventive measures [17]. A meta-analysis of ARI among under-five children based on 12 Indian studies conducted since 1994, estimated incidence rates between 2.4 to 7.4 episodes per child per year and also highlighted the lack of community-based studies on etiology of ARI from India [18]. The few existing studies on etiology of pneumonia have all been hospital based [19, 20].

In order to fill these evidence gaps in the epidemiology of community-acquired ARI in India, we established community-based surveillance among a cohort of rural children up to 10 years of age, with a plan to extend this cohort to a vaccine-testing platform. We chose to include a cohort until 10 years of age, as there is very little information on ARIs in the 5–10 year age group. This paper describes this on-going surveillance platform and presents the preliminary findings from its first year of data collection.

## Methods

The study is funded by a co-operative agreement between the United States Centers for Disease Control and Prevention (CDC) and the All India Institute of Medical Sciences (AIIMS), New Delhi. The Institutional Ethics Committee of the AIIMS, New Delhi approved the study, with an institutional reliance by the US CDC Institutional Review Board. A written informed consent was obtained from the either parent of the children for children under 7 years of age and from both parents and children for children aged 7 or more years.

**Fig. 1:**
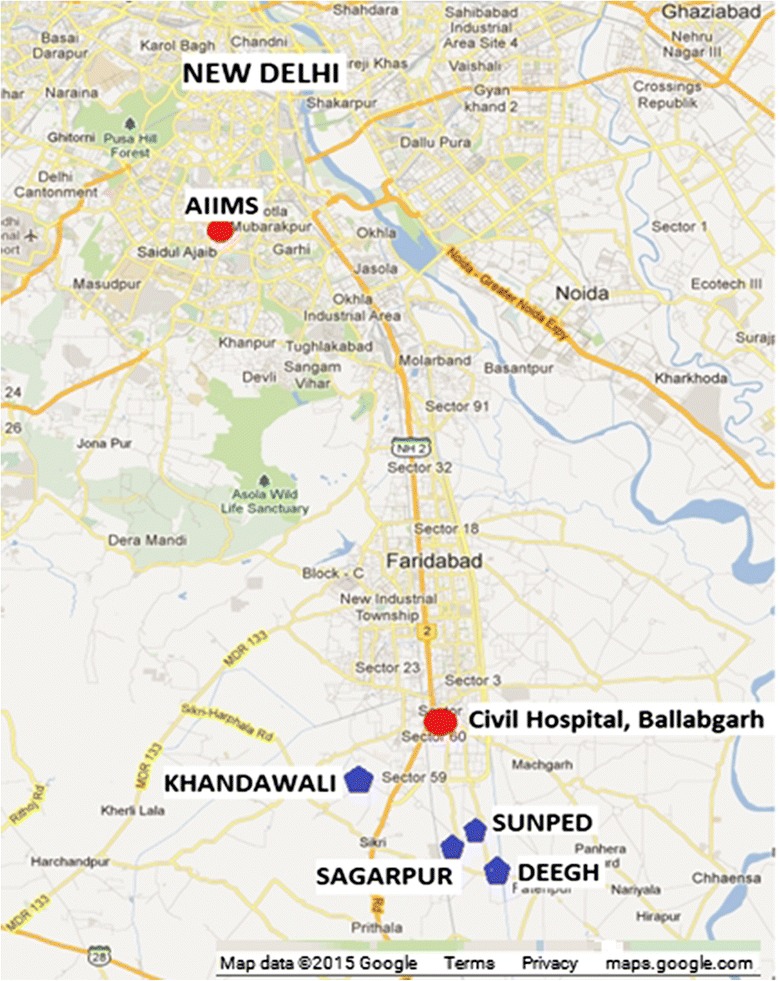
Map of the study area

The study area consists of four villages, Sunped, Sagarpur, Deegh and Khandawali in the rural Ballabgarh Block of Faridabad district, in the north Indian state of Haryana. The villages are situated approximately 40 km south of New Delhi. (Figure 1) In this region, there are three seasons: winter (October to February), summer (March to June) and monsoon (July to September). These communities are typically agrarian with creeping urbanization from the nearby Ballabgarh town. The study villages are served by the Mohna Primary Health Centre (Sunped, Sagarpur and Deegh) and Panheda Khurd PHC (Khandawali), located at a distance of about 15 to 20 Km. Ballabgarh town is about 5 km away and has a secondary level government health facility as well as a multitude of private health facilities. In each village, Auxiliary Nurse Midwives (ANM) provide primary medical facilities such as immunization and maternal care.

From May to July 2012, a house-to-house census was carried out in these villages. A household was defined as people sharing the same kitchen. The census data were fed into a computerised database system to establish a listing of all individuals in the study villages. Based on the census data, all households with eligible children (children < 10 years of age whose families had been a resident for at least six months) were identified. Either the parent or a guardian of the child was explained the objectives of the study, and the benefits and harms of participation in the local language and their written consent was obtained.

Active weekly surveillance for ARI in this dynamic cohort was started on 13th August 2012 after an initial one week pilot to streamline the workflow. The final data for analysis for the first year was from 13th August 2012 to 9th August 2013. Follow-up continued until a child - i) reached 10 years of age ii) withdrew consent; iii) migrated out of the study area; iv) or died. In addition to weekly surveillance, vital events such as births, deaths, migrations and marriages are regularly updated. Verbal autopsy is carried out to identify the cause of death among these children using validated tools [21] an annual census is carried out to update any missed vital events, and to make note of any changes in households which also helps in rescheduling the visit schedule of surveillance workers.

### Acute respiratory infections

**Fig 2:**
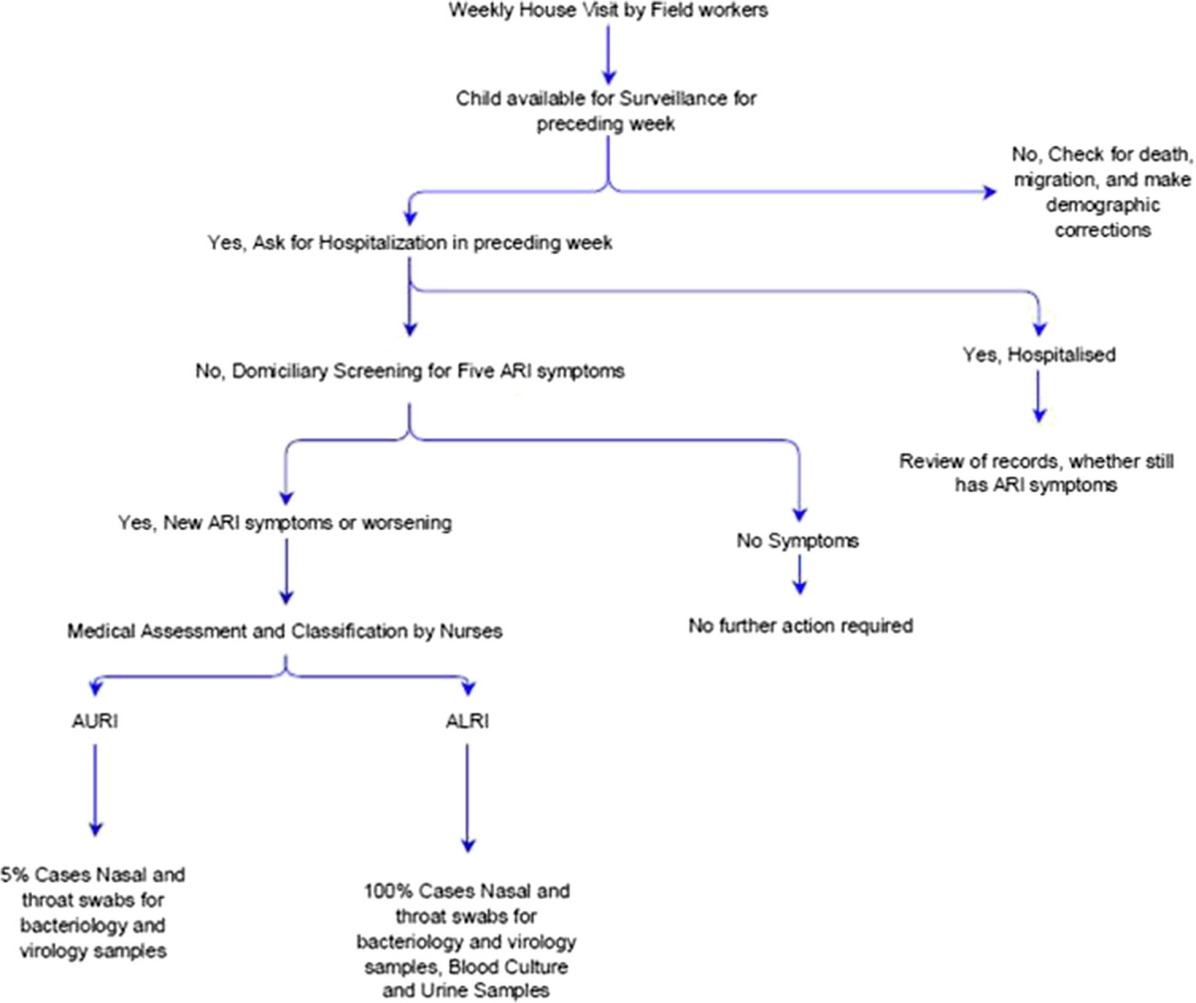
Flow chart depicting the surveillance platform

Each child is followed up weekly at home by trained workers who ask for presence of one or more of five symptoms of ARI (cough, sore throat, nasal congestion, earache/discharge, breathing difficulty) (Fig. 2). If a new symptom is present or has worsened from the previous week, nurses are informed who take a detailed clinical history and perform examination (respiratory rate, peripheral capillary oxygen saturation, axillary temperature among others) of all children identified as having an ARI. Thereafter, children are assigned a diagnosis as per Integrated Management of Childhood and Neonatal Illnesses (IMNCI)/Integrated Management of Adolescent and Adult Illnesses (IMAI) guidelines [22, 23] (Table 1). A classification of “possible serious bacterial infection”, “severe pneumonia” or “very severe disease and pneumonia” under IMNCI & IMAI are jointly considered as Acute Lower Respiratory Infections (ALRI) and non- serious bacterial infections and no pneumonia are grouped together as Acute Upper Respiratory Infections (AURI). No chest auscultation or radiological confirmation of pneumonia is performed. Nurses provide appropriate medical advice and basic treatment including antibiotics to all sick children as recommended under national guidelines. In severe cases, referral to higher medical facilities is advised and facilitated. In two of the study villages, study clinics are run once a week in existing buildings to provide outpatient consultations. Surveillance workers also note any hospitalisations of enrolled children during their weekly visits. Doctors then examined them, either in the hospital or at their homes after discharge, and confirmed ARI- related hospitalisations based on available documentation. In short, surveillance workers identify children with respiratory symptoms and hospitalization during weekly home visits, children with respiratory symptoms are clinically examined by nurses to classify the severity and doctors review all hospitalizations to identify respiratory infection related admissions.

**Table 1:** Acute Respiratory Infection (ARI) case definition and classification

Age group	Condition items	Category	Nurse grading of ARI
<2 months	Any one of following danger signs: Convulsions; Fast breathing (60 breaths/ min); Severe chest in drawing; Nasal flaring; Grunting; Bulging fontanel; 10 or more skin pustules or a big boil; If axillary temperature 37.5 °C or above (or feels hot to touch) or temperature less than 35.5 C (or feels cold to touch); Lethargic or unconscious; Less than normal movements	Possible serious bacterial infection	Acute Lower Respiratory Infection
	None of the above mentioned signs or symptoms and presence of respiratory symptoms (except sore throat)	Non-serious bacterial infection	Acute Upper Respiratory Infection
	Presence of any one of the following general danger signs: convulsions; Inability to drink or feed or breastfeed; Lethargy or unconsciousness; Vomits everything; and/or any one of the following:	Severe pneumonia or very severe disease	Acute Lower Respiratory Infection
	Chest in-drawing		
	Stridor in calm child		
2 months	Fast breathing (with age-specific cut-off rates)	Pneumonia	Acute Lower Respiratory Infection
10 years	2 months - 12 months: 50 breaths/min		
	12 months - 5 years: 40 breaths/min		
	5–10 years: 30 breaths/min		
	No signs of pneumonia or very severe disease but presence of any of the symptoms of ARI	No pneumonia -cough or cold	Acute Upper Respiratory Infection

ARI was defined as occurrence of a new onset or worsening of one or more of the following five symptoms: cough, sore throat, nasal congestion, earache or discharge, breathing difficulty

The quality of collected surveillance data is checked by health supervisors who revisit 10 % sample of the surveyed households, check for completion of forms in the field. Most inconsistencies were minor and were a result of change in the respondent. In addition, the supervisors compiled daily reports, enquired about births, deaths and migration in the villages, conducted verbal autopsy on children who died, a sub-sample of which were crosschecked by physicians.

### Biological samples

Nasal and throat swabs were collected from a random sample of 5 % of AURI cases and from all ALRI cases for virology and bacteriology tests. Samples were also collected in a similar manner from age-matched asymptomatic controls from the cohort members of neighbouring houses for each ALRI case to estimate carriage rates for different etiologic agents. Urine samples for antigen detection by Binax Test for Streptococcus pneumoniae were collected from ALRI cases and controls more than one year of age. Blood sample was taken from ALRI cases for culture and sensitivity testing (using BacT/ALERT® 3D, biomerieux, France). For bacteriology testing, one nasal (for <1 year olds) or throat (for >1 year olds) sample by Dacron swab was collected and immediately placed into bacterial transport media (STGG - Skimmed milk, tryptone, glycerol and glucose) on ice for transportation. The nasal and oropharyngeal samples were transported on ice in triple sealed containers. Urine and blood culture samples were transported at room temperature. Samples were transported to the microbiology laboratory for further culture for bacteriological agents and RNA extraction and RealTime-PCR testing for viruses.

### Measurement of risk factors for ARI

Towards the end of year one, along with a census we carried out two assessments: i) the socio-economic status of all households in the study area based on possession of household consumer items and based on the wealth index used in the National Family Health Survey (NFHS) [24] and ii) measurement of ARI risk factors among study children including child health indicators (anthropometry, clinical examination for signs of iron and vitamin deficiency, information on breastfeeding practices, immunisation, vitamin A supplementation and school going status) and household environmental assessment (cooking fuel used, ventilation, overcrowding and second hand exposure to tobacco smoke).

### Data management

Demographic data of the four study villages and data from ARI screening carried out by surveillance workers were entered into a MySQL database, and data on clinical assessments (from nurses) were entered separately. Standardized data collection forms were used to provide consistent data recording of clinical, laboratory and interview data. Prior to data entry, forms were reviewed for completeness, consistency and logic by both clinical and data management personnel. A small sample of data entered by data entry operators were cross-checked with surveillance forms completed by field workers and nurses. Data were periodically checked by descriptive statistics to identify outliers. Regular encrypted backups of the data have been made and backup logs maintained.

All analyses were conducted in Stata 12/IC (StataCorp, 2011). Person-time (child-weeks) was used as denominators and each new episode of respiratory infection was taken after a 24-h symptom free interval. All incidence rates were calculated per child-year. Here we present findings from the first year of surveillance.

## Results

In the study villages, ground water is the predominant source of drinking water (61.8 %); about a quarter of households practice open defecation, two-thirds (69.1 %) use bio-fuels for cooking and 61 % have a tobacco smoker in the house. On average each house had 1.75 children below 10 years of age (Table 2).

**Table 2:** Household characteristics of houses in Surveillance platform at Ballabgarh for Acute Respiratory Tract Infections SuBhARTI cohort, 2012–13 (*n* = 2292)

Household characteristics	Value
% of houses with	
Land ownership	42.2
Head of household with agriculture related occupation	18.1
Ground water as main source of drinking water	61.8
Piped water in own house	26.2
Access to water seal latrine	74.7
Wood/cow dung as main Fuel used for cooking	69.1
Tobacco smoker in the HH	60.5
Mean (Standard Deviation) number of	
Persons per household	5.97 (2.61)
Children ≤ 10 years per household	1.75 (1.54)
Sleeping rooms per household	1.89 (0.98)

**Fig 3:**
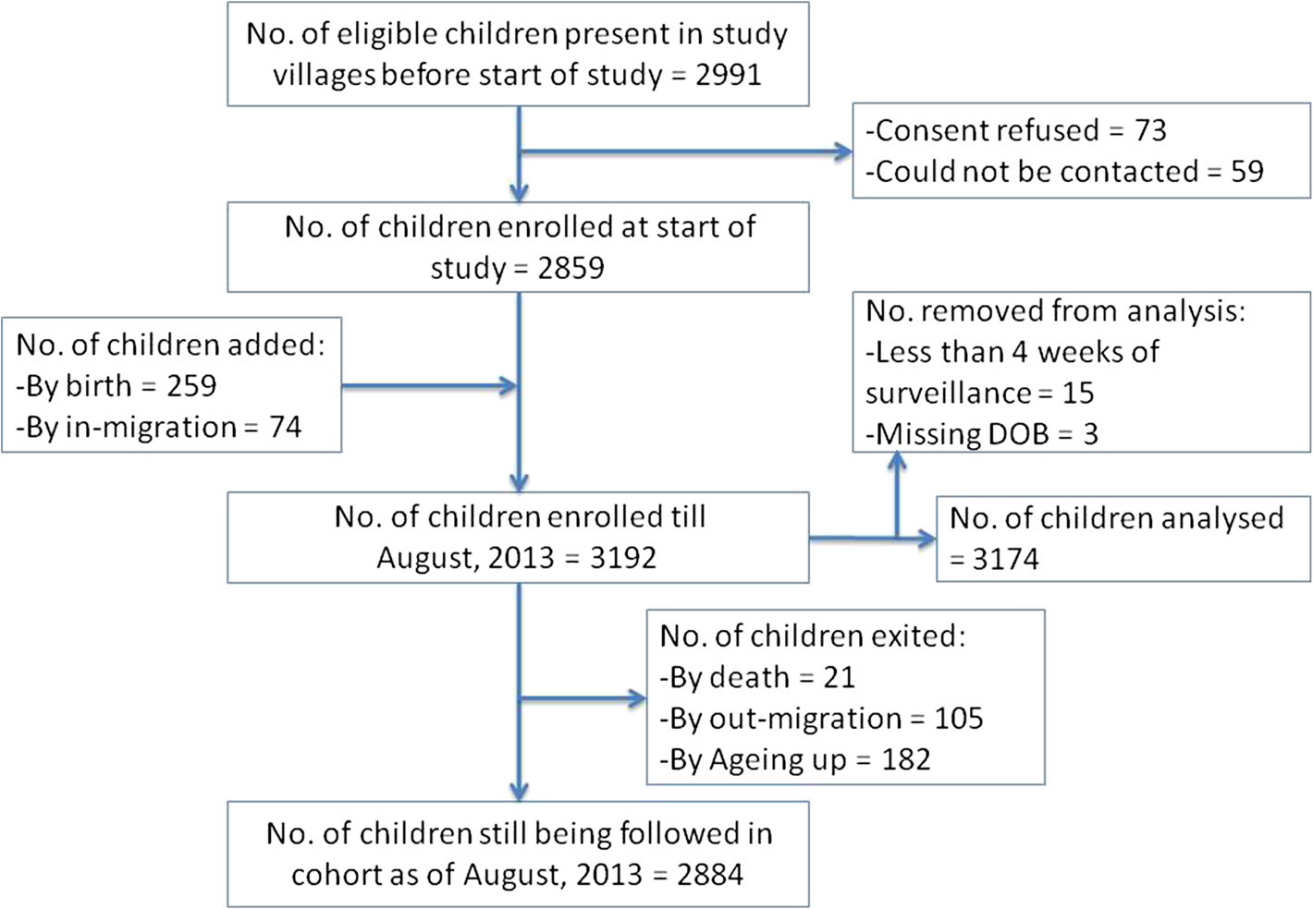
Flow chart of enrollment and follow-up of the cohort

At the start of surveillance on 13th August 2012, there were 2859 children (refusal rate of 2.4 %) enrolled in the cohort, by the end of first year a total of 3192 children had been enrolled in the cohort with 2884 still remaining in the cohort at the end of year 1 (Fig. 3). 182 children reached 10 years of age; none withdrew consent; 105 children migrated out of the study area and 21 died during this period. During the one-year period, 259 newborns and 74 immigrated children were added to the cohort. Those children who were enrolled in the study but followed up for less than four weeks (*n* = 15) and those without complete information (*n* = 3) were excluded, leaving 3174 children for this analysis.

The cohort was comprised of slightly more boys (52.4 %) than girls (47.6 %), and their age distributions were similar (Table 3). Together these children contributed 137,836 child weeks of surveillance (52.9 % by boys and 47.1 % by girls). More than half of the children (53.8 %) underwent surveillance for 48 weeks or more in the first year (Table 3). There were no major differences in surveillance coverage by seasons.

**Table 3:** Age at enrolment and follow up of cohort (*n* = 3174 children)

Characteristics	Boys		Girls		Total	
	Nos.	%	Nos.	%	Nos.	%
Age at enrolment						
First year of life	259	(15.6)	253	(16.7)	512	(16.1)
1–5 years	659	(39.7)	611	(40.4)	1270	(40.0)
5–10 years	744	(44.8)	648	(42.9)	1392	(43.9)
Total	1662	(52.4)	1512	(47.6)	3174	(100)
Weeks of surveillance						
Up to 27 weeks	159	(9.6)	176	(11.6)	335	(10.6)
28 to 35 weeks	97	(5.8)	107	(7.1)	204	(6.4)
36 to 43 weeks	200	(12.0)	212	(14.0)	412	(13.0)
44 to 47 weeks	275	(16.5)	240	(15.9)	515	(16.2)
48 to 52 weeks	931	(56.0)	777	(51.4)	1708	(53.8)

### ARI incidence

The surveillance workers identified a total of 16,524 episodes of ARI. Of these, 15,607 (94.5 %) were assessed and classified into age appropriate ARI categories, 363 episodes were reported to be asymptomatic at the time of nurse visit (mainly due to the change of respondents) and 554 episodes could not be assessed due to absence of the child.

The ARI incidence in 0–10 years of age was 5.88 (95 % CI: 5.79–5.98) per child-year with minimal gender difference (Table 4). The ALRI incidence in the under-five age group was 0.43 (0.39–0.49) per child-year among boys and 0.31 (0.26–0.35) per child-year among girls with a total incidence rate of 0.37 (0.34–0.40) per child-year. The highest incidence of ARI related hospitalization was among boys in 29 days to 1-year age group (82.2 per 1000 child-year). Among the total under-fives this was 15.0 per 1000 child years with girls 3.6 times lower rates of ARI-related hospitalization (Table 4). Four deaths due to pneumonia were reported in this cohort in the first year.

**Table 4:** Burden of respiratory infections among under-ten children by age and sex, 2012–13 (*n* = 3174 children)

Age Group	Weeks of surveillance		Acute Respiratory Infections				Acute Lower Respiratory Infections				ARI related Hospital Admissions			
			ARI				ALRI							
			Episodes		Incidence*		Episodes		Incidence*		Episodes		Incidence*	
	Boys	Girls	Boys	Girls	Boys	Girls	Boys	Girls	Boys	Girls	Boys	Girls	Boys	Girls
0 to 28 days	107	103	9	13	4.37	6.56	1	0	0.49	0	0	0	0	0
29 days to 11 months	5 690	5 412	1 159	1 042	10.59	10.01	125	86	1.14	0.83	9	2	0.0822	0.0192
12 to 35 months	15 300	14 319	2 517	2 349	8.55	8.53	138	75	0.47	0.27	5	2	0.0169	0.0072
36 to 59 months	15 288	13 255	1 934	1 566	6.58	6.14	40	34	0.14	0.13	2	0	0.0068	0
5 to 10 years	36 546	31 816	2 659	2 359	3.78	3.86	31	18	0.04	0.03	0	2	0	0 .0032
Under - 5 years	36 385	33 089	5 619	4 970	8.03 (7.80 –8.20)	7.81 (7.60–8.00)	304	195	0.43 (0.39–0.49)	0.31 (0.26–0.35)	16	4	0.0228 (0.0139–0.0371)	0.0062 (0.0017–0.0161)
Under −10 years	72 931	64905	8278	7 329	5.90 (5.80–6.00)	5.87 (5.70–6.00)	335	213	0.24 (0.21–0.27)	0.17 (0.15–0.20)	16	6	0.0114 (0.0065–0.0185)	0.0048 (0.0018–0.0105)

*Incidence expressed as episodes per child year; Figures in parentheses are 95 % confidence intervals

## Discussion

Although there are some smaller cohort studies and a few cross-sectional studies in India, this is the first large-scale and comprehensive study of community-based ARI with more than 3000 children under surveillance [25–31]. Our preliminary results are similar to other studies among under-five children who have reported an incidence of pneumonia between 0.25 to 0.50 episodes per child per year in the South-Asia region [17, 32]. The sex differentials in hospitalization due to ARI in South—Asia has been commented by Nair et al. previously as well [33].

To the best of our knowledge, this is the first study to provide community-based ARI incidence among 5–10 year olds in India. Our sample size was calculated to establish a minimum expected agent-specific incidence of 0.035 episodes per child per year (10 % of 0.35 pneumonia incidence) with a relative precision of 20 and 95 % confidence. Our clinical definition of ARI was broad as compared to studies identified in reviews of ARI both in India and else where in under-five children which might have resulted in a slight overestimate of AURI and potentially could include non-infectious causes of cough as well [18, 34, 35]. However, this was not the case with ALRI as trained nurses, who were closely monitored by physicians, used standard classification guidelines for ALRI ascertainment. The continuous nature of the weekly surveillance platform minimized the possibility of missing ARI episodes regardless of duration, as long as the child was present in the study area. The rigorous quality control measures and the continuity of data collection irrespective of holidays add to the credibility and robustness of our estimates.

The weaknesses in this study include the classification of ARI as AURI and ALRI was determined by clinical assessments and did not include auscultation or radiology and could have resulted in some misclassification. Due to the time lag between birth and enrolment, surveillance was missed in the early neonatal period. It is possible that repeated contacts with the family members might have resulted in overestimation of ARI due to reporting of mild symptoms that would have otherwise been unnoticed. The rigorous surveillance could also have resulted in early recognition and treatment seeking, preventing progression of AURI to ALRI, potentially leading to an underestimation of ALRI in the community. While it is difficult to decide an appropriate recall period and most studies have used a two week recall period, we feel that weekly surveillance represents the best trade-off between missing due to recall and development of fatigue due to repeated visits [36, 37].

## Conclusion

The cohort established as a part of this collaboration will provide critical information on respiratory infections epidemiology in rural India. The preliminary results show that ARI impose a significant burden on the children of this cohort. Further analysis of the data would help us elucidate the risk factors, social inequities related to this disease. We have just initiated a vaccine trial using live attenuated influenza vaccine and social contact mixing studies in the established cohort to generate more evidence on transmission dynamics and prevention of respiratory infection in this population.

## Abbreviations

AIIMS: All India Institute of Medical Sciences
ANM: Auxillary Nurrse Midwife
ARI: Acute Respiratory Tract Infection
ALRI: Acute Lower Respiratory Tract Infection
AURI: Acute Upper Respiratory Tract Infection
CDC: Centers for Disease Control and Prevention
GAPP: Global Action Plan for Prevention and Control Of Pneumonia
IMNCI/IMAI: Integrated Management of Childhood and Neonatal Illnesses/Integrated Management of Adolescent and Adult Illnesses
NFHS: National Family Health Survey
PHC: Primary Health Centre
SuBhARTI: Surveillance platform at Ballabgarh for Acute Respiratory Tract Infections
